# A possible link between BDNF and mTOR in control of food intake

**DOI:** 10.3389/fpsyg.2014.01093

**Published:** 2014-09-25

**Authors:** Nobuyuki Takei, Kazuo Furukawa, Osamu Hanyu, Hirohito Sone, Hiroyuki Nawa

**Affiliations:** ^1^Department of Molecular Neurobiology, Brain Research Institute, Niigata University, NiigataJapan; ^2^Department of Hematology, Endocrinology and Metabolism, Faculty of Medicine, Niigata University, NiigataJapan

**Keywords:** BDNF, mTOR, mTORC1, food intake regulation, body weight, AMPK, nutrients

## Abstract

Food intake is intricately regulated by glucose, amino acids, hormones, neuropeptides, and trophic factors through a neural circuit in the hypothalamus. Brain-derived neurotrophic factor (BDNF), the most prominent neurotrophic factor in the brain, regulates differentiation, maturation, and synaptic plasticity throughout life. Among its many roles, BDNF exerts an anorexigenic function in the brain. However, the intracellular signaling induced by BDNF to control food intake is not fully understood. One candidate for the molecule involved in transducing the anorexigenic activity of BDNF is the mammalian target of rapamycin (mTOR). mTOR senses extracellular amino acids, glucose, growth factors, and neurotransmitters, and regulates anabolic reactions response to these signals. Activated mTOR increases protein and lipid synthesis and inhibits protein degradation. In the hypothalamus, mTOR activation is thought to reduce food intake. Here we summarize recent findings regarding BDNF- and mTOR-mediated feeding control, and propose a link between these molecules in eating behavior.

## INTRODUCTION

Several lines of evidence indicate that neurons in the hypothalamus sense nutrient sufficiency. These neurons are regulated by many factors involved in feeding control, such as leptin (Morton et al., 2006). The roles of many other feeding-related peptides, both orexigenic and anorexigenic, have also been extensively studied. For example, clear obesity and leanness phenotypes are observed in knockout mice lacking pro-opiomelanocortin (POMC; a precursor of α-melanocyte-stimulating hormone (MSH); Yaswen et al., 1999) and melanin-concentrating hormone (MCH; Shimada et al., 1998), respectively. Recently, another factor involved in regulation of feeding and metabolic regulation in the brain has come into the spotlight: brain-derived neurotrophic factor (BDNF; Ooi et al., 2012; Rios, 2013; Vanevski and Xu, 2013; Marosi and Mattson, 2014).

## BRAIN-DERIVED NEUROTROPHIC FACTOR

Brain-derived neurotrophic factor is the most prominent neurotrophic factor in the central nervous system. Indeed, BDNF and its cognate high-affinity receptor, TrkB, are widely expressed in the brain from development to adulthood. BDNF promotes differentiation, maturation, and survival of neurons, and plays important roles in synaptic plasticity through the activation of TrkB, a receptor tyrosine kinase (Nawa and Takei, 2001; Park and Poo, 2013). TrkB-expressing (i.e., BDNF-responsive) neurons are distributed in the arcuate nucleus (ARC), paraventricular nucleus (PVN), lateral hypothalamus (LH), ventromedial nucleus (VMH), and dorsomedial nucleus (DMN) of the hypothalamus (Yan et al., 1997).

## BDNF AND REGULATION OF FOOD INTAKE

The first evidence that BDNF is involved in body weight control came from a rather serendipitous result. While assessing the neuroprotective effects of neurotrophins, Lapchak and Hefti (1992) found that chronic intracerebroventricular (ICV) infusion of BDNF in adult rats after fimbrial lesion reduced body weight. Subsequent systematic experiments also revealed that ICV injection of BDNF suppressed appetite and promoted weight loss in rats (Pelleymounter et al., 1995). The second clear line of evidence that BDNF plays crucial role in food intake comes from studies of genetically manipulated mice. Mice heterozygously deleted for the gene encoding BDNF (*Bdnf*^+/-^) produce half of the wild-type level of BDNF protein and exhibit a severely obese phenotype due to overeating (Lyons et al., 1999; Kernie et al., 2000). Furthermore, brain-specific deletion of *Bdnf* (Rios et al., 2001), deletion of dendritic BDNF mRNA (Liao et al., 2012), shRNA-mediated knockdown of BDNF using a viral vector (Unger et al., 2007), and a hypomorphic allele of *Trkb* that expresses only a quarter of TrkB all result in hyperphagia, obesity, and metabolic imbalances such as hyperglycemia (Xu et al., 2003).

Genotype–phenotype interactions indicate that BDNF–TrkB signals also play important roles in weight control in humans. For instance, a *de novo* missense mutation of the *TRKB* gene, Tyr722Cys, which leads to a defect in downstream signaling, was identified in an 8-year-old male who presented with hyperphagia, severe obesity, and developmental delay (Yeo et al., 2004). Similarly, patients with Wilms’ tumor, aniridia, genitourinary anomalies, and mental retardation (WAGR) syndrome, who have a truncation of chromosome 11, exhibit hyperphagia and obesity. Analysis of the genomes of WAGR patients revealed that they are heterozygous for deletion of *BDNF*. Patients with *BDNF* haploinsufficiency were all obese, whereas only 20% of WAGR patients without *BDNF* deletion were obese (Han et al., 2008). A common single-nucleotide polymorphism (SNP) of *BDNF*, G196A, produces the amino acid substitution Val66Met in the prodomain. The Val66Met mutant exhibits defects in intracellular trafficking and activity-dependent release of mature BDNF (Egan et al., 2003). A genome-wide association study linked this SNP to susceptibility to obesity in humans (Beckers et al., 2008; Speliotes et al., 2010; Waterhouse and Xu, 2013). Likewise, in an experimental model, G196A knock-in mice exhibit increased body weight (Chen et al., 2006). Because these mutations in humans are all genomic, not somatic, the effect of BDNF deficiency on metabolic abnormalities may arise from systemic and/or developmental activities. Indeed, it has been proposed that BDNF contributes to metabolism in peripheral organs (Nakagawa et al., 2000; Hanyu et al., 2003). However, brain- or hypothalamus-specific deletion or knockdown of BDNF induces overeating and obesity. These results suggest that BDNF acts directly on the hypothalamic circuit that regulates food intake and metabolism, thereby controling body weight. In addition, BDNF injection into the brain can rescue the obese phenotype of mutant mice. These results indicate that BDNF exerts its anorexic action in an acute, temporally specific manner, and that the effects of loss of BDNF on feeding behavior are not the result of developmental defects in neural circuits. BDNF also acts on midbrain dopaminergic neurons, which are involved in hedonic eating related to the reward/addiction system (Cordeira et al., 2010; Alsiö et al., 2012). Thus, this system may contribute to overeating in individuals carrying these mutations. Future studies should investigate these issues in greater detail.

Previous studies of eating behavior have focused on extracellular cues and neural circuits in the hypothalamus, but have not looked as closely at intracellular signaling mechanisms. Considering the acute effect of BDNF on food intake, we wondered what signaling molecules play major roles in BDNF-mediated feeding control. Mammalian target of rapamycin (mTOR), a kinase that governs metabolism in peripheral cells, has attracted attention as a regulator of food intake through the brain (Cota, 2009; Wiczer and Thomas, 2010; Howell and Manning, 2011; André and Cota, 2012). BDNF is a major activator of mTOR in neurons (Takei et al., 2001, 2004; Inamura et al., 2005) therefore, we hypothesize that the anorexigenic action of BDNF is mediated by mTOR in neurons. Before elaborating on this idea, we will provide a brief introduction of mTOR and its signaling pathways.

## MAMMALIAN TARGET OF RAPAMYCIN

**FIGURE 1:**
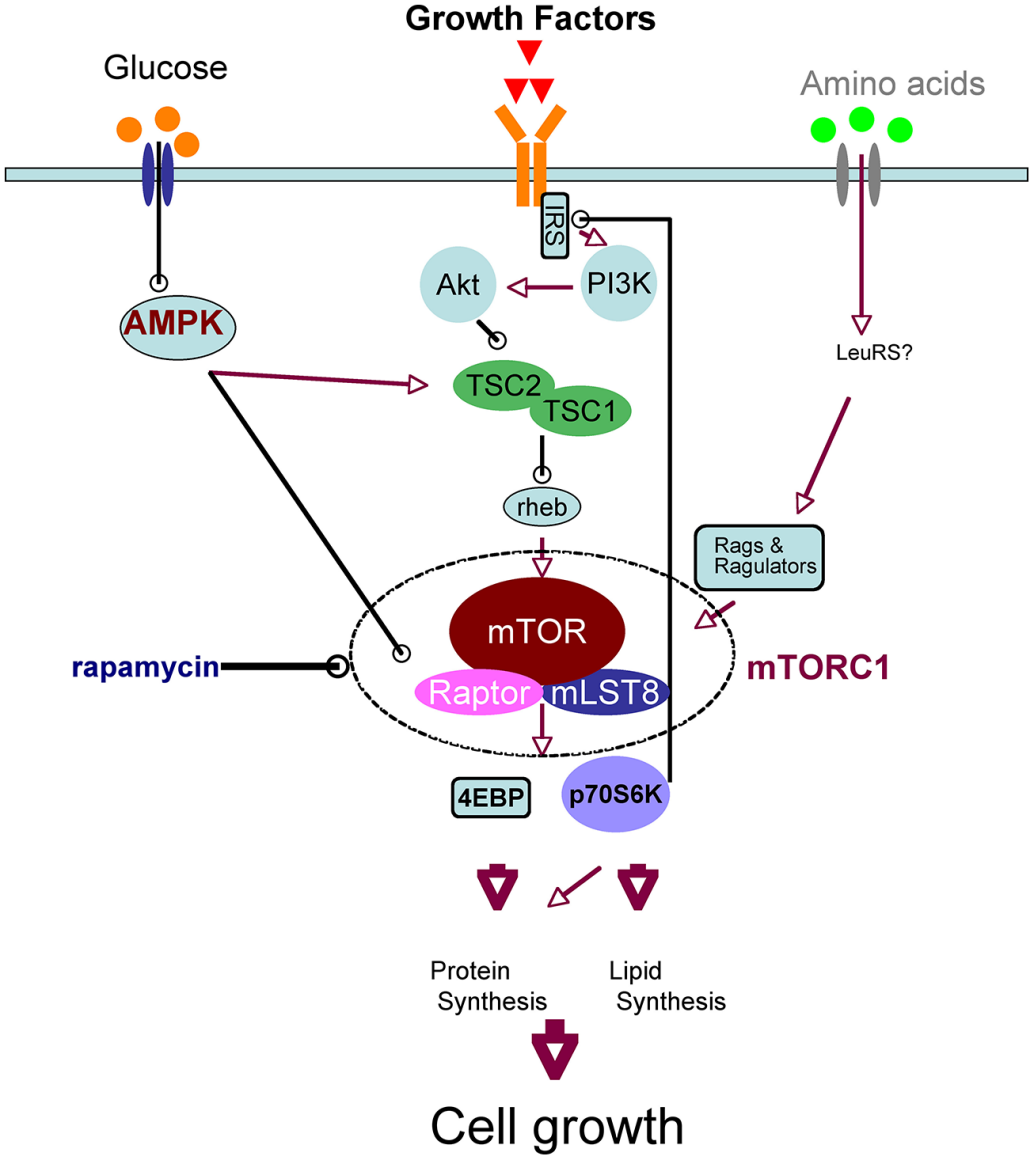
**Simplified schematic of upstream and downstream mTORC1 signaling**.

Mammalian target of rapamycin is the mammalian ortholog of yeast TOR, which is the target molecule of rapamycin, an anti-fungal and immunosuppressant compound. mTOR is a serine/threonine kinase that forms two complexes, mTOR complex 1 (mTORC1) and 2, which have different molecular partners. mTORC1 is a key component of the nutrient-sensing network that controls cellular metabolism: it integrates various extracellular cues, such as nutrients (amino acids and glucose) and growth factors, and it regulates various biochemical processes, including translation, autophagy, transcription, and lipid biosynthesis. These biochemical reactions induce anabolic states and thereby promote cell growth. The signaling pathways upstream and downstream of mTORC1 have recently been elucidated (Laplante and Sabatini, 2012; Takei and Nawa, 2014); we have provided a simplified schematic of neuronal mTOR signaling in **Figure 1** Leucine, taken up by the system L-amino acid transporter, activates mTORC1 (Ishizuka et al., 2008). Growth factors such as BDNF (Takei et al., 2001, 2004; Inamura et al., 2005), insulin (Lee et al., 2005), and insulin-like growth factor (Quevedo et al., 2002) activate the phosphoinositide 3-kinase (PI3K)/Akt pathway through their tyrosine kinase receptors in neurons. Akt directly phosphorylates tuberous sclerosis complex 2 (TSC2), a suppressor of Rheb that activates mTORC1. When glucose levels are sufficient, AMP-activated protein kinase (AMPK) activity decreases, and thus mTORC1 becomes active (Dash et al., 2006). These inputs converge on mTORC1; therefore, the availability of amino acids and/or glucose is essential for growth factor-mediated mTORC1 activation (Hara et al., 1998; Ishizuka et al., 2013). mTORC1 phosphorylates eukaryotic initiation factor 4E-binding protein (4EBP) and thereby stimulates translation. In addition, phosphorylation of p70S6 kinase (p70S6K) by mTORC1 also promotes translation and lipid biosynthesis, whereas phosphorylation of ULK1 inhibits autophagy. All of these processes increase total protein and lipid levels in the cell and thereby increase cellular mass (Laplante and Sabatini, 2012; Takei and Nawa, 2014). It should be noted that mTORC1 signaling is regulated by a feedback mechanism: mTORC1 and p70S6K phosphorylate and inactivate insulin receptor substrate (IRS; Tremblay and Marette, 2001; **Figure 1**), which interacts receptor tyrosine kinases such as insulin receptor and TrkB (Yamada et al., 1997). Thus, prolonged activation or overactivation of mTORC1 results in desensitization of this signaling cascade; this is thought to be one mechanism of insulin resistance (Tremblay and Marette, 2001).

## mTOR AND REGULATION OF FOOD INTAKE

In the brain, the first hint of a relationship between food intake and mTORC1 signaling was provided by the involvement of AMPK on feeding. AMPK is a cellular fuel gage that senses the AMP/ATP ratio and turns off metabolic pathways that consume ATP (Kemp et al., 2003). Because AMPK itself plays a key role in metabolism, the regulation of hypothalamic AMPK was initially investigated independently of mTOR (Minokoshi et al., 2004, 2008). Leptin, insulin (Minokoshi et al., 2004), and cilliary neurotrophic factor (CNTF; Steinberg et al., 2006) reduce AMPK activity in the hypothalamus similarly to re-feeding after fasting; thus, these molecules suppress food intake and thereby reduce body weight. By contrast, adiponectin (Kubota et al., 2007), ghrelin, and AICAR (Andersson et al., 2004), a pharmacological activator of AMPK, stimulate AMPK activity, and thus increase food intake and body weight. Moreover, adenovirus-mediated expression of dominant-negative (DN) AMPK reduces food intake and body weight, whereas expression of constitutive-active (CA) AMPK increases them (Minokoshi et al., 2004). Recent findings regarding the signaling network (**Figure 1**) suggest that regulation of food intake by AMPK may converge on the mTORC1 system in the hypothalamus. AMPK activates TSC2, a suppressor of mTORC1, and thereby inhibits mTORC1 (Inoki et al., 2003). In addition, AMPK phosphorylates Raptor, a scaffold protein of mTORC1, also leading to inhibition of mTORC1 (Gwinn et al., 2008). Thus, like rapamycin, AMPK activation suppresses mTORC1 and increases food intake.

Direct evidence that mTORC1 signaling might be coupled with feeding has been reported (Cota et al., 2006). Similar to the action of leptin, ICV injection of leucine activates mTORC1 and reduces food intake. Moreover, the effects of both leucine and leptin in the brain on mTORC1 signaling and food intake are counteracted by rapamycin, a specific mTORC1 inhibitor. Fasting and re-feeding also affect mTORC1 signaling in neurons in the hypothalamus. It remains unclear which area of the hypothalamus, as well as which types of neurons, participates in mTORC1-mediated regulation of food intake. The answer to this question might be provided by precise analysis using methods such as immunohistochemistry with phospho-specific antibodies to mTOR network molecules. Studies of mTORC1 signaling on food intake using genetically modified mice have yielded seemingly paradoxical consequences. Global deletion of p70S6K (s6k^-/-^), a downstream signaling molecule of mTORC1, protects against diet-induced obesity (Um et al., 2004). By contrast, injection of an adenovirus vector carrying DN-p70S6K into the mediobasal hypothalamus increases food intake and body weight, whereas overexpression of CA p70S6K reduces both parameters (Blouet et al., 2008; Ono et al., 2008). Furthermore, conditional deletion of *Tsc1*, an upstream suppressor of mTORC1 in hypothalamic neurons (and in beta cells of the pancreas), induces hyperphagic obesity, and hypothalamic POMC neuron-specific deletion of *Tsc1* results in the same phenotype (Mori et al., 2009). Because *Tsc1* deletion induces mTORC1 activation, this phenomenon seems contradictory to the anorexic effect of mTORC1.

**FIGURE 2:**
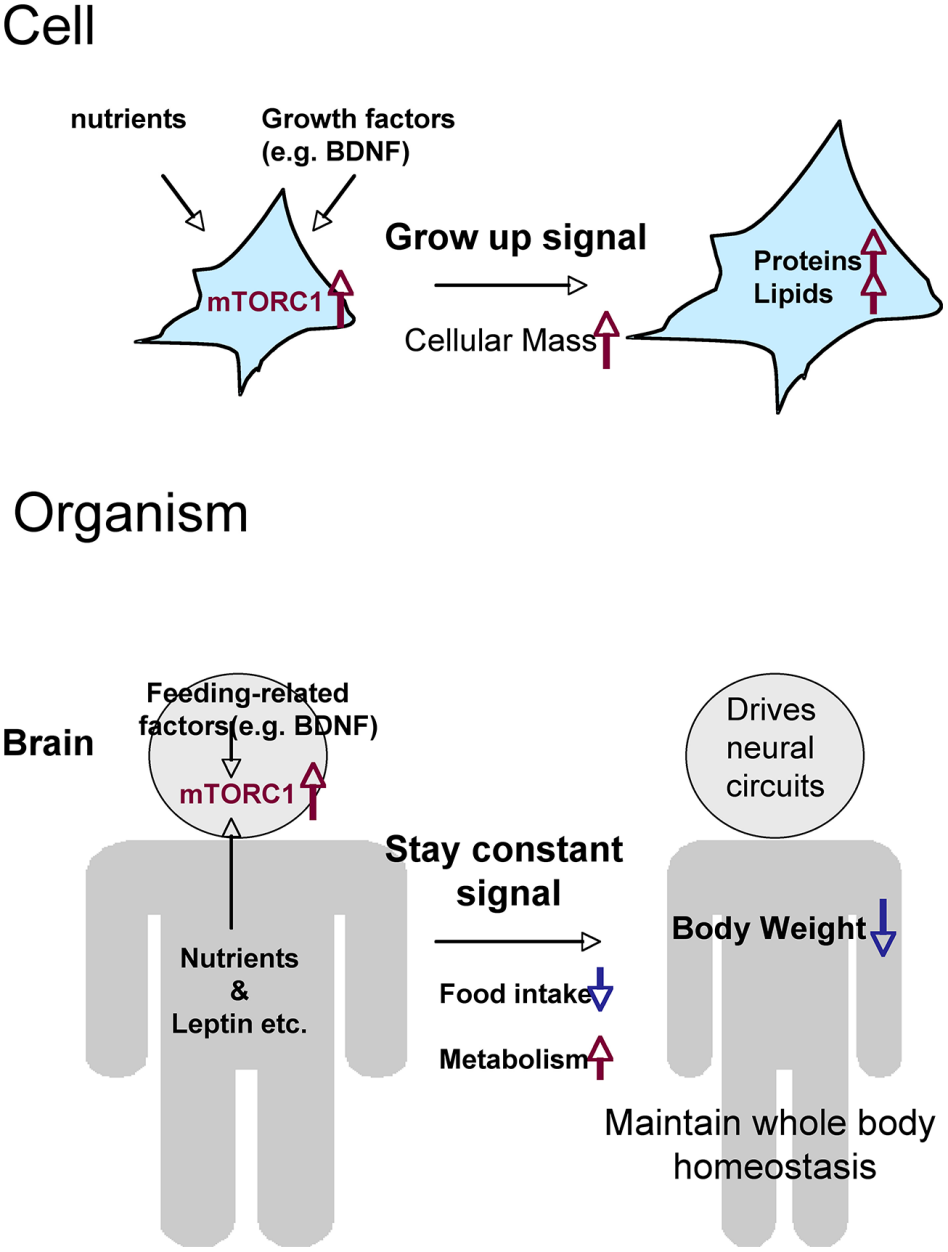
**Hypothetical roles of mTORC1 on metabolism and food intake, comparing the cellular and organismal levels.** Through the activation of mTORC1, cells that receive nutrients and growth factors grow by increasing total levels of proteins and lipids. Cells (neurons) in the brain activate mTORC1 in response to nutrients and a variety of feeding-related factors. The resultant signals give the order to stop eating and maintain whole-body homeostasis.

Two issues complicate our understanding of mTORC1’s action on food intake and weight control: the negative-feedback mechanism and the difference between the peripheral (or systemic) and central (brain) activities of mTORC1. Prolonged activation of mTORC1 signaling induced by gene knockouts of upstream or downstream molecules may cause inactivation of mTORC1 in the hypothalamus via negative-feedback. Thus, for example, knockout of *Tsc1* may cause orexigenic rather than anorexigenic effects due to long-lasting feedback suppression of mTORC1. Precise biochemical analysis of mTORC1 signaling in these animals may help to resolve this paradox. Systemic mTORC1 pathway activation induces cell growth in many organs, and thereby increases body weight; this may explain why global knockout of p70S6K suppresses weight gain (Blouet et al., 2008; Ono et al., 2008). Nevertheless, because the organism must maintain whole-body homeostasis, it is quite likely that phasic activation of mTORC1 in hypothalamic neurons drives an anorexic state. In other words, mTORC1 senses the “satiety” signal in the brain in order to maintain an appropriate body weight (**Figure 2**).

## BDNF AND mTOR

Amino acids, glucose, and growth factors induce mTORC1 activation in the hypothalamus and reduce food intake. In addition to leucine and leptin, CNTF (Cota et al., 2008) and bone morphogenic protein 9 (BMP9; Townsend et al., 2012) also suppress food intake. Culture studies have revealed that these molecules elicit mTORC1 signaling in neuronal cells. Leucine activates mTORC1 through the system L-amino acid transporter in primary cultured neurons (Ishizuka et al., 2008), whereas CNTF induces the phosphorylation of STAT3 via mTORC1 in neuroblastoma cells (Yokogami et al., 2000). These phenomena are similar to the effects of BDNF on both mTORC1 signaling and food intake. On the cellular level, BDNF is a potent activator of mTORC1 in neurons, and it stimulates anabolic responses such as protein synthesis (Takei et al., 2001, 2004). Although there is as yet no direct evidence, it is very likely that the anorexigenic action of BDNF is mediated by mTORC1 activation in the hypothalamus. Importantly, BDNF-mediated mTORC1 activation is limited by glucose availability (Ishizuka et al., 2013). Because glucose and amino acids are indispensable for the maintenance of homeostasis, this observation suggests the existence of a safeguard system in which glucose sufficiency overrides other mTORC1-activating stimuli in neurons. Therefore, it is necessary to obtain direct evidence that the anorexigenic action of BDNF is really mediated by mTORC1 signaling, either via the use of mTOR inhibitors such as rapamycin or knockdown of mTORC1 components in the hypothalamus. In addition, it is also important to determine which types of neurons in the hypothalamus are actually responsible for mTORC1-mediated feeding control. The use of a unique promoter-driven Cre-mouse (such as POMC-Cre) to make conditional knockout in mTOR or mTORC1 components in certain hypothalamic neurons may be useful in this regard.

In unicellular organisms and at the single-cell level in metazoans, nutrient uptake and subsequent mTORC1 activation lead to cell growth (i.e., increase in cellular mass and/or proliferation). In multicellular organisms, the brain regulates food intake to maintain whole-body homeostasis. Thus, mTORC1 in hypothalamic neurons senses many complex signals, both from the periphery (e.g., glucose, amino acids, insulin, leptin, and ghrelin) and from neural networks within the brain (e.g., via peptides and BDNF; **Figure 2**). The mechanisms by which the brain controls feeding behavior are complex, but mTORC1 may represent a cellular crossroads for the regulation of food intake and metabolism by nutrients and other inputs.

## Conflict of Interest Statement

The authors declare that the research was conducted in the absence of any commercial or financial relationships that could be construed as a potential conflict of interest.
